# Maximal Voluntary Force Strengthened by the Enhancement of Motor System State through Barely Visible Priming Words with Reward

**DOI:** 10.1371/journal.pone.0109422

**Published:** 2014-10-02

**Authors:** Yudai Takarada, Daichi Nozaki

**Affiliations:** 1 Faculty of Sports Sciences, Waseda University, Tokorozawa, Saitama, Japan; 2 Graduate School of Education, The University of Tokyo, Bunkyo-ku, Tokyo, Japan; Radboud University Nijmegen, Netherlands

## Abstract

The topic of unconscious influences on behaviour has long been explored as a means of understanding human performance and the neurobiological correlates of intention, motivation, and action. However, what is relatively unknown is whether subconsciously delivered priming stimuli, with or without rewards, can affect individuals’ maximum level of force produced with their best effort. We demonstrated using transcranial magnetic stimulation that barely visible priming of an action concept, when combined with a reward in the form of a consciously visible positive stimulus, could alter the state of the motor system. In accordance with this neurophysiological alteration, the prime-plus-reward stimuli significantly increased the hand-grip force level of maximum voluntary contraction with little conscious awareness. This is the first objective evidence that the barely conscious presence of a behavioral goal can influence the state of the motor system and arouse latent ability for human force exertion.

## Introduction

In everyday life, we perform many of our actions without thinking, automatically and effortlessly. Research has documented the ability of healthy people to pursue behavioral goals unconsciously [1]. Moreover, associating such goals with a positive reward signal can enhance motivation to produce powerful actions independently of any reported motivation to attain the goals [2]. In fact, it is already known that subliminal priming with action words can facilitate or inhibit motor performance and can activate or deactivate motor representations [3], [4]. Also, such positive stimuli-induced reward signals are processed by the basal ganglia, in the ventral pallidum to be precise, resulting in motivating people to increase the effort they invest in behaviors or to recruit the resources necessary for maintaining behaviors [5].

However, the manner in which subconsciously delivered priming stimuli with rewards can alter the state of the pyramidal motor system is not yet fully understood. Here, we investigated the influence of the aforementioned unconscious goal pursuit on the motor system by examining the motor evoked potentials (MEP) produced by transcranial magnetic stimulation (TMS) applied over the hand area of the contralateral primary motor cortex (M1) (Experiment 1). We demonstrated that the motor system was more excitable when subliminal priming words were rewarded by positive words.

In a previous study by Aarts et al. [2], submaximal force exertion was strongly enhanced by subliminal priming combined with positive reward words. Not only the force level but also reaction time, rate of increase in applied force, and total effort (mean force over time) were increased. However, what is unknown is whether subconsciously delivered priming stimuli, with or without rewards, could affect individuals’ maximum levels of force produced with their best effort.

Human maximal voluntary force involves both neural and morphological factors. All morphological factors being equal, maximal voluntary force is usually limited by the participant’s capacity to activate motor units. Maximal voluntary force is also generally limited by neural inhibition, and the activation of muscle fibers may be inhibited in about half of participants even when they are asked to exert force with their maximal volition [6]. This inhibition has been related to the supraspinal “drive” operating on the motor units [6]. Indeed, Ikai and Steinhaus proposed in a pioneering study [7] that maximal voluntary force is limited by psychological inhibiting factors, based on experimental results showing that such force was enhanced by manipulations such as the sound of a gunshot or a shout during maximal exertion efforts. These results indicate that force exertion in the motor system is relatively inhibited and that there is a latent ability for producing additional force hidden in ordinary force exertion. We hypothesized that the enhancement of the motor system by subliminal priming combined with reward, which was demonstrated by Experiment 1, could also enhance maximal voluntary force (Experiment 2).

## Materials and Methods

### Ethics Statement

The experimental procedures were in compliance with relevant laws and institutional guidelines and were approved by the Human Research Ethics Committee of the Faculty of Sport Sciences, Waseda University (approval number 2012-062).

### Participants and Procedure

Two experiments examined the influence of barely conscious goal pursuit on motor evoked potentials (MEPs) in the flexor carpi ulnaris (FCU) in response to TMS in a resting phase (Experiment 1) and during the application of maximal right hand-grip force (maximal voluntary contraction, or MVC) (Experiment 2). Seventy-nine healthy Japanese right-handers (evaluated using the Edinburgh Handedness Inventory) [8] participated in the study after providing both written and oral informed consent.

### Experiment 1: Influence of barely conscious goal pursuit on the motor system

In this experiment, we used TMS to examine how barely conscious goal pursuit altered the state of the motor system. Nineteen healthy volunteers (16 males and three females; mean age ± S.D.  = 20.5±1.9 years) participated. Pregnant women were excluded to avoid the unknown risks of fetal exposure to TMS.

Based on the experimental procedure reported by Aarts et al. [2], the following three conditions were used: control condition, priming condition, and priming-plus-reward condition. Participants experienced all three conditions in random order with a 15-min interval. The numbers of participants for possible in six combinations of the execution order were as follows: (control, priming, priming-plus-reward) 4; (control, priming-plus-reward priming) 2; (priming, control, priming-plus-reward) 2; (priming, priming-plus-reward, control) 5; (priming-plus-reward, control, priming) 3; (priming-plus-reward, priming, control) 3. On each trial, participants were either shown priming words related to exertion or were shown random letters, followed by positive or neutral words. To investigate the excitability of the motor cortex, TMS was applied to the left primary motor cortex 1.5 s after the positive or neutral word disappeared. Thus, 50 MEPs were obtained for each condition.

To examine the influence of these conditions on actual motor behavior, we also asked the participants to squeeze the handgrip-force apparatus three times with their right hand for 5 s each time (inter-trial interval = 6 s) at the end of each condition.

### Experiment 2: Influence of barely conscious goal pursuit on MVC

In this experiment, we investigated whether barely conscious goal pursuit contributed to changes in maximal force exertion. The same experimental conditions were used as in Experiment 1. Sixty healthy participants (28 males and 32 females; mean age ± S.D.  = 19.6±1.2 years) were assigned randomly to one of the three conditions (i.e., each participated in only one condition; *n* = 20 for each group). Before and after participants engaged in the trials for their respective conditions, they were asked to exert MVC of hand-grip force with the right hand three times for 5 s each time, with a 6-s inter-trial interval.

### Priming procedure

On the basis of a pilot study (*n* = 80), we selected five Japanese words pertaining to the goal of physical exertion [“
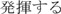
” (“exert” in English), “
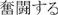
” (“struggle”), “
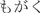
” (“work hard”), “
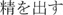
” (“energize”), and “
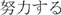
” (“strive”); *Mean* = 7.98 on a 9-point scale], five positive adjectives [“
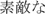
” (“nice”), “
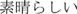
” (“great”), “
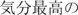
” (“fantastic”), “
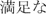
” (“satisfactory”), and “
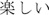
” (“enjoyable”); *Mean* = 7.62], and five neutral adverbs [“
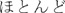
” (“almost”), “
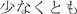
” (“at least”), “
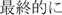
” (“finally”), “
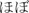
” (“nearly”), and “
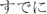
” (“already”); *Mean* = 4.84].

In the priming-plus-reward condition, for 25 of the 50 trials, barely visible presentation of one of the five exertion words was followed by fully visible presentation of one of the five positive words. For the remaining 25 trials, barely visible presentation of a random letter string was followed by fully visible presentation of one of the five neutral words. Thus, in this condition, the barely visible exertion primes were always paired with positive words. In the priming condition, the exertion primes were paired with neutral words (25 trials), and the random letter strings were paired with positive words (25 trials). Thus, in this condition, although exertion primes and positive words were both displayed, they were never paired with each other. In the control condition, only random letter strings were used as primes and were paired with positive words on 25 trials and with neutral words on 25 trials; barely visible exertion words were never displayed. In this way, the viewing of positive and neutral words was balanced at 25 trials each for all conditions. The order of possible stimulus pairs was randomized within each condition.

**Figure 1:**
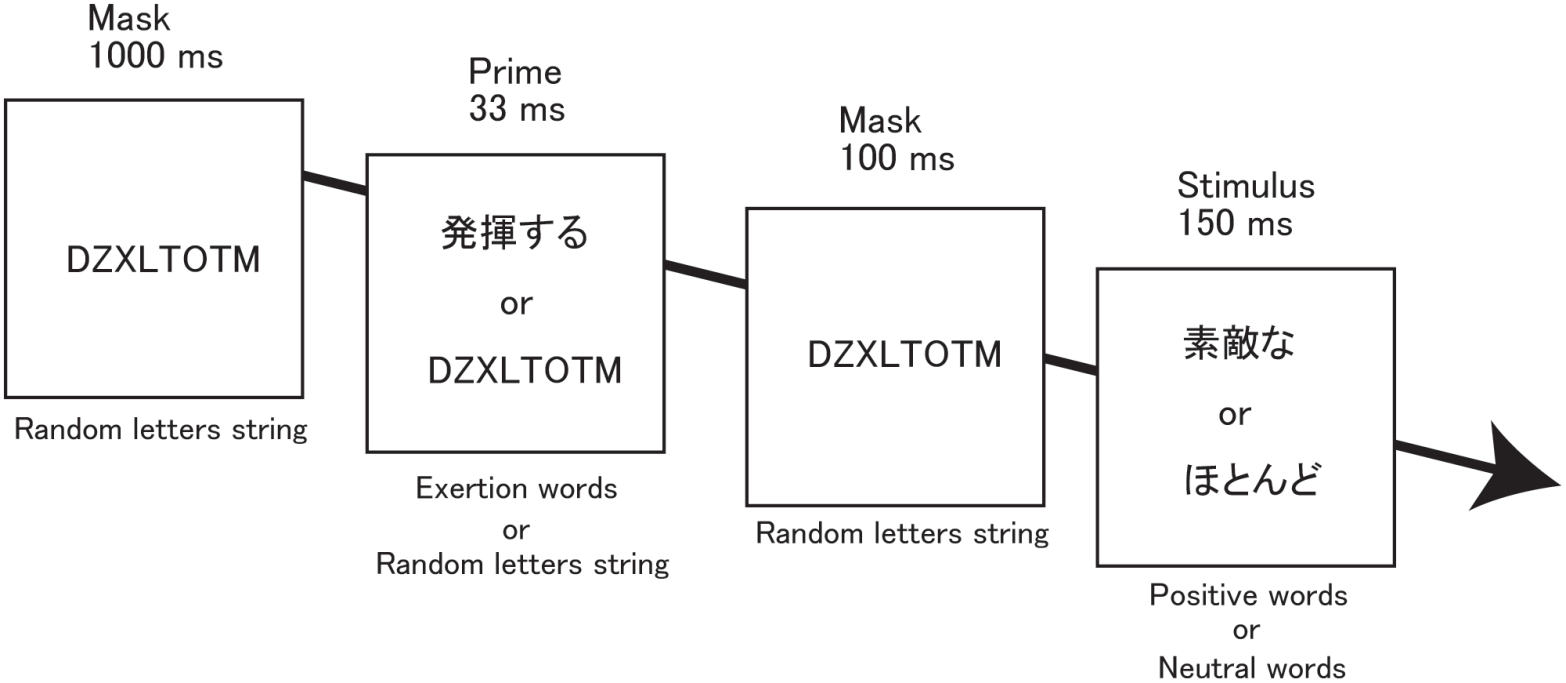
Priming procedure. In the priming-plus-reward condition, the subliminal barely visible exertion primes were always paired with positive words. In the priming condition, although exertion primes and positive words were both displayed, they were never paired with each other. In the control condition, subliminal barely visible exertion words were never displayed. The order of possible stimulus pairs was randomized within each condition. Exertion, positive, and neutral words were Japanese. Each trial in each condition began with a 1000-ms presentation of a random eight-letter string (e.g., DZXLTOTM) as a forward mask. This was followed by the subliminal barely visible prime, displayed for 33 ms. A random letter string was again displayed for 100 ms as a backward mask, after which a consciously visible word was presented for 150 ms. Occasionally, a dot was presented for 33 ms (it was visible because of the absence of a backward mask), either above or below the neutral or positive word.

Each trial in each condition began with a 1000-ms presentation of five different kinds of a string of eight pseudorandom letters (DZXLTOTM, YSTZBXTU, VCFTHYPC, CBEXGTVY, and ZTAWYDBH) as a forward mask (Fig. 1). This was followed by the barely visible prime, displayed for 33 ms. One randomly-selected random letter string among those five was again displayed for 100 ms as a backward mask, after which a consciously visible word was presented for 150 ms. Occasionally, a dot was presented for 33 ms (visible because of the absence of a backward mask), either above or below the neutral or positive word. Participants were instructed to indicate whether they had seen a dot, to bring the post-masked barely visible primes to their attention. Trials occurred every 3.5 s within each condition. We used a 60-Hz CRT screen to display the words, and the experimental procedure was created with software designed for psychological experiments (Inquisit 3 Desktop Edition, Millisecond Software, Seattle, WA, USA).

### Hand-grip force measurement

Force was measured using a hand-grip device (KFG-5-120-C1-16, Kyowa Electronic Instruments, Tokyo, Japan). After viewing all 50 stimulus pairs, participants were asked to squeeze the hand-grip device with the right (dominant) hand when “squeeze” (in Experiment 1) or “squeeze with full power” (in Experiment 2) appeared on the display, and to stop squeezing when those words disappeared. The squeeze instruction was displayed for 5 s. The squeeze task was repeated three times with a 6-s inter-trial interval. After the three trials in Experiment 1, participants were also asked to rate, on a category-ratio scale (CR-10 scale) [9], how hard they had tried to squeeze the device. A rating of 1 signified ‘very light’, and 10 signified ‘very, very hard’. In Experiment 1, reaction time (time from the display of the “squeeze” instruction to the production of hand-grip force), rate of force (rate of increase in applied force =  the first peak in force curve/arrival time of the first peak), and total effort (mean force over time) were quantified as motor actions (behaviors) from the obtained force curve. In Experiment 2, the mean peak force levels across the three trials were defined as MVC.

### MEP measurement

In Experiment 1, 1.5 s after the positive or neutral words disappeared, TMS was delivered via a stimulator (SMN-1200, Nihon Koden, Tokyo, Japan) using a figure-8 coil (7 cm inner diameter, 11 cm outer diameter, YM133B). The coil was positioned over the finger area of the left M1, which was determined using the lowest resting motor threshold (RMT) for the FCU muscle in the right hand; MEPs in FCU muscle with peak-to-peak amplitudes of approximately 100 µV were induced in at least 5 of 10 trials when participants were totally relaxed with their eyes closed [10]. Coil position was stabilized throughout the experiment with a coil stand made from multiple products (Manfrotto Distribution KK, Tokyo, Japan). The optimal scalp position of M1 was marked directly on the scalp with a black magic marker. The RMT ranged from 50 to 85% of maximum stimulator output, at which the averaged waveform of MEP (an average of 10 recordings evoked by TMS) was defined as the baseline MEP. Stimulus intensity was set at 110% of the RMT.

During MEP recording in Experiment 1, participants were asked to remain at a resting state. Surface electromyograms were obtained with bipolar surface silver electrodes (bandpass, 15 Hz–10 kHz). For each condition, the peak-to-peak amplitude of the averaged MEP across 50 trials was calculated. The background electromyogram (EMG) was calculated as the integral of the rectified EMG signal during the period 20 ms before TMS.

### Statistical analysis

We undertook a one-way analysis of variance (ANOVA) to test the differences in reaction time, rate of force, total effort, MEP, and % change of MVC across the three experimental conditions. If necessary, the Greenhouse-Geisser correction was applied to adjust degrees of freedom for violations of the sphericity assumption. Post-hoc analysis used the Bonferroni correction for multiple comparisons. Statistically significant differences in MVC between the “baseline” and “post” phases were investigated using paired t-tests for each experimental condition. A significance level of *p*<0.05 was chosen for all tests.

## Results and Discussion

### Experiment 1: Influence of barely conscious goal pursuit on the motor system

**Figure 2:**
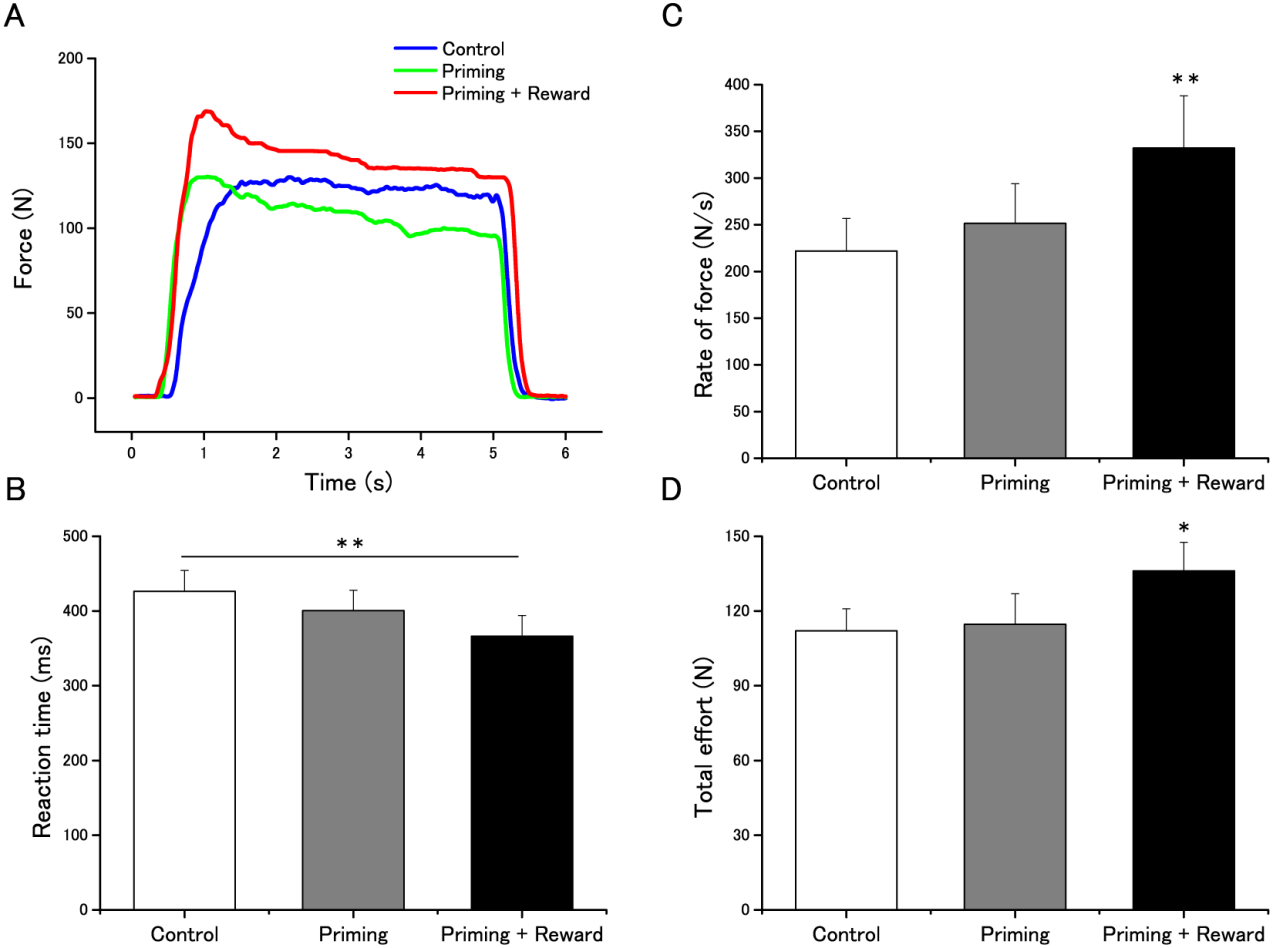
Effects of barely conscious goal pursuit on motor action. A: Typical recordings of hand-grip force after three experimental conditions. B, C, D: Reaction time (B), Rate of force (C), and Total effort (D) for the three conditions. Data are expressed as mean ± SEM. The asterisks in panel B indicate statistically-significant differences between the control and the priming-plus-reward conditions, and the asterisks in panels C and D indicate statistically -significant differences compared with other conditions (*p<0.05, **p<0.01).

Several effects on behavior were observed. Reaction time [Fig. 2A, B; F_(2, 36)_  = 6.97; MSE = 0.002; *p* = 0.003; Effect size: η^2^ = 0.279], rate of force (rate of increase in applied force) [Fig. 2A, C; F_(1.25, 22.5)_  = 12.13; MSE = 0.031; *p* = 0.001; Effect size: η^2^ = 0.403], and total effort (mean force over time) [Fig. 2A, D; F_(2, 36)_  = 4.56; MSE = 0.003; *p* = 0.017; Effect size: η^2^ = 0.202] differed according to condition. Post-hoc analyses revealed a significantly higher rate of force {comparison with control condition, [*t*(18) = −3.29, *d* = 0.60; *p*<0.001]; comparison with priming condition, [*t*(18) = −3.52, *d* = 0.41; *p* = 0.003]; comparison between priming and control conditions, [*t*(18) = −1.55, *d* = 0.19; *p* = 0.62]} and greater total effort {comparison with control condition, [*t*(18) = −2.61, *d* = 0.44; *p* = 0.025]; comparison with priming condition, [*t*(18) = −2.23, *d* = 0.42; *p* = 0.049]} in the priming-plus-reward condition than in the other conditions; there was no significant difference between the priming and control conditions [*t*(18) = 0.88, *d* = 0.046; *p* = 1.00]. Reaction time in the priming-plus-reward condition was significantly shorter than in the control condition [*t*(18) = −3.31, *d* = 0.55; *p* = 0.002] but not in the priming condition [*t*(18) = −1.95, *d* = 0.32; *p* = 0.127]; there was no significant difference between the priming and control conditions [*t*(18) = −1.09, *d* = 0.22; *p* = 0.35]. The priming effects on motor preparation have been already observed in a previous study [2], and may be based on the ideomotor principle [11].

In an independent test of the conscious perception of the primed exertion words, an additional and different sample of participants (mean age ± S.D.  = 22.7±2.3 years, *n*  = 20) was subjected to the priming-plus-reward treatments. Specifically, over the 50 trials participants were asked to indicate whether they saw a word related to physical exertion or not. The post-masked subliminal primes of exertion were attended to but not reportable. The mean percentage of correct responses was 50.3 (SD = 5.9).

These results suggest that participants in the priming–plus-reward condition started squeezing earlier (Fig. 2A, B) than in the control condition and increased their force faster (Fig. 2A, C) and with more total effort (Fig. 2A, D) than those in the control and priming conditions did. However, levels of subjective effort did not change in the control (5.47±0.30), priming (5.15±0.37), or priming-plus-reward conditions (5.47±0.25), [F_(2, 36)_  = 0.69; MSE = 0.90; *p* = 0.506; Effect size: η^2^ = 0.037], suggesting that the observed differences in motor action were caused mainly by barely conscious information processing. These results are generally consistent with other recent findings [2], [12].

**Figure 3:**
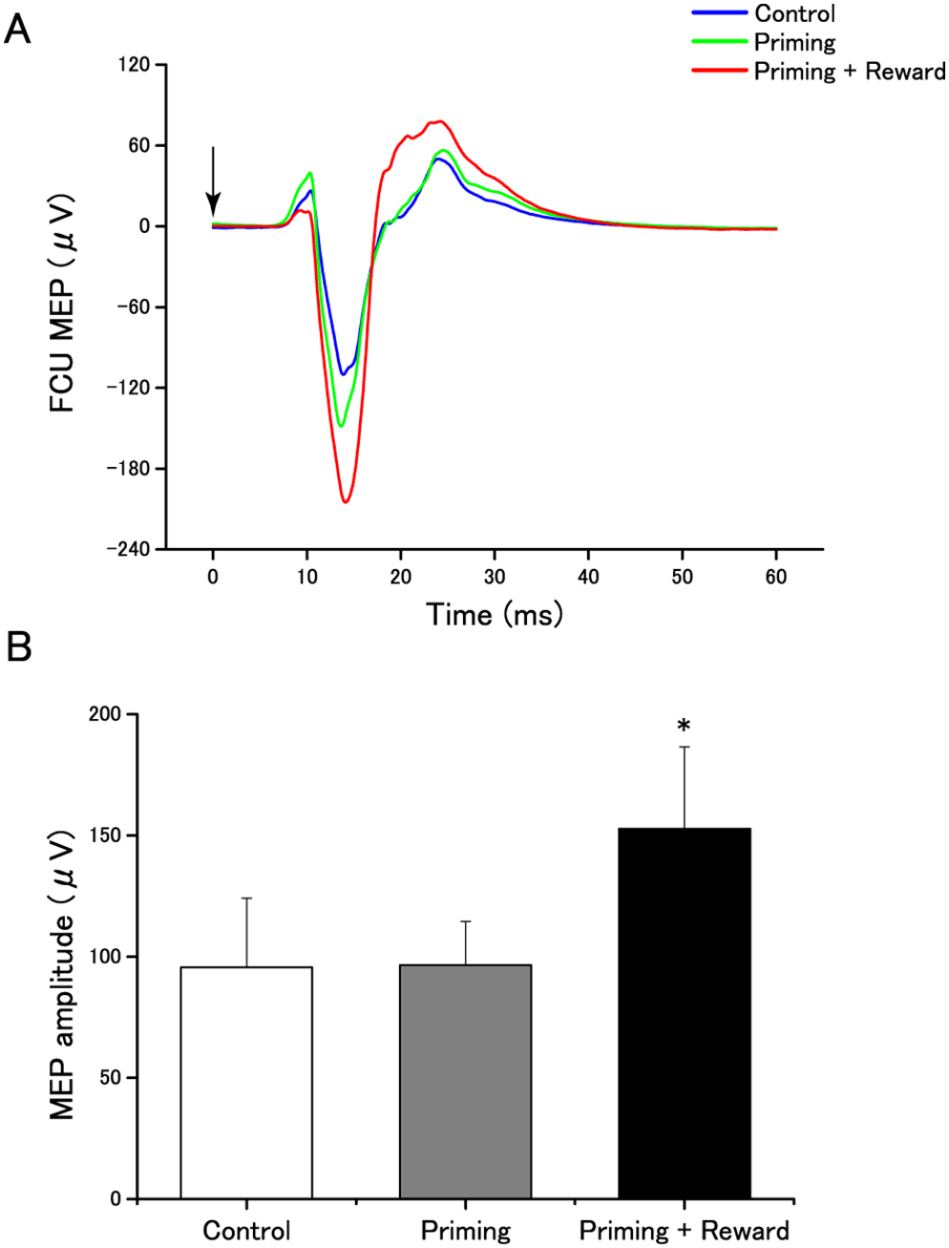
Effects of barely conscious goal pursuit on MEP amplitude. A: Typical recordings of the average MEP of all 50 trials for FCU during three experimental conditions in a singleparticipant. Arrows indicate the timing of TMS. B: Amplitudes of MEPs for the three experimental conditions. Data are expressed as mean ± SEM. Asterisks indicate statistically -significant differences compared with other conditions (*p<0.01).

Baseline MEPs in the three conditions were statistically equivalent [F_(2, 36)_  = 0.32; MSE = 296.9; *p* = 0.72; Effect size: η^2^ = 0.018], but surprisingly, marked differences were observed (Fig. 3A) during the three experimental conditions [F_(2, 36)_  = 7.34; MSE = 2777.1; *p* = 0.002; Effect size: η^2^ = 0.290]. Post-hoc analyses confirmed larger amplitudes of MEPs in the priming-plus-reward condition than in the other two conditions {comparison with control condition, [*t*(18) = −4.06, *d* = 0.51; *p* = 0.006]; comparison with priming condition, [*t*(18) = −2.36, *d* = 0.56; *p* = 0.007]; Fig. 3B; comparison between priming and control conditions, [*t*(18)  = 1.78, *d* = 0.011; *p* = 1.00]}. The increase in the average MEP over all 50 trials was also observable in the averaged MEPs of the first 16 consecutive trials (MEP1), the second 17 consecutive trials (MEP2), and the last 17 consecutive trials (MEP3). Background electromyogram (EMG) data showed no significant differences among conditions, [F_(2, 36)_  = 0.828; MSE = 30.2; *p* = 0.45; Effect size: η^2^ = 0.044]; therefore, a change in background EMG cannot explain the difference in MEPs across conditions. Taken together, the behavioral and MEP results showed that barely visible priming stimuli paired with reward stimuli enhanced corticospinal excitability, leading to more forceful and effortful voluntary motor action with barely conscious awareness. Therefore, we next asked whether such barely visible priming stimuli contributed to increases in MVC (Experiment 2).

### Experiment 2: Influence of barely conscious goal pursuit on MVC

**Figure 4:**
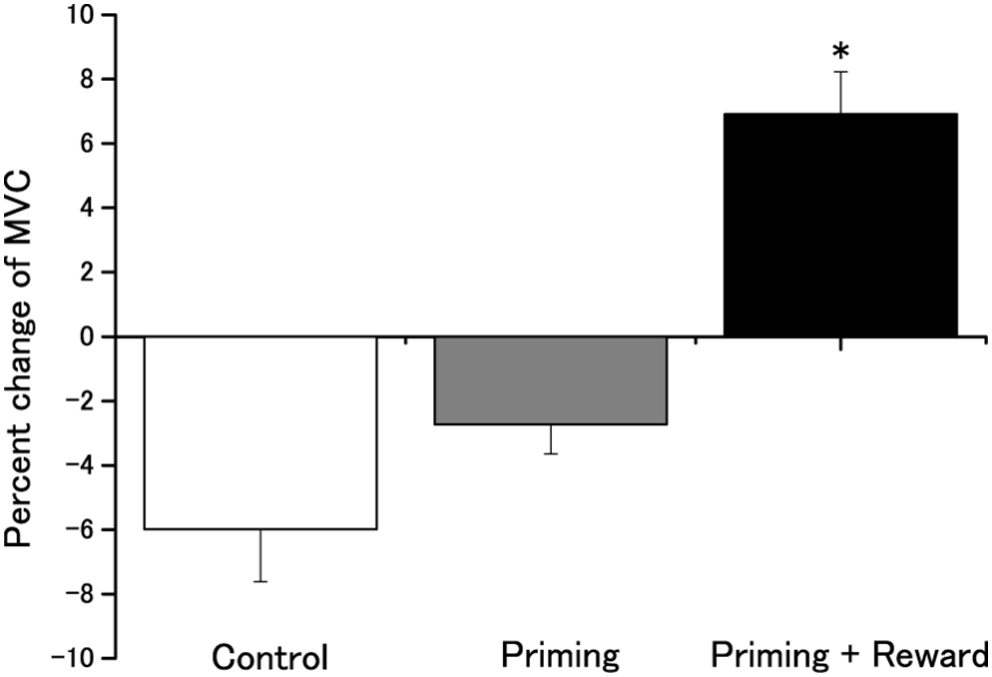
Effects of a barely conscious motivator on MVC. MVC for the three conditions. Data are expressed as a percentage of baseline values (mean ± SEM). An asterisk indicates statistically-significant differences compared with other conditions (*p<0.01).

Percent change in MVC differed across conditions [F_(2, 57)_  = 25.9; MSE = 34.6; *p*<0.001], and post-hoc analyses revealed a significantly larger percent change in MVC in the priming-plus-reward than in the other conditions {comparison with control condition, [*t*(38) = −5.23, *d* = 1.95; *p*<0.001]; comparison with priming condition, [*t*(38) = −4.67, *d* = 1.90; *p*<0.001]} (Fig. 4). There was also no significant difference between in the priming and control conditions [*t*(38) = −1.55, *d* = 0.55; *p* = 0.250]. MVC values in the “baseline” phase did not differ significantly across the three conditions [F_(2, 57)_  = 0.60; MSE = 0.022; *p* = 0.550]. Paired t-tests showed that MVC in the “post” phase was significantly greater than in the “baseline” phase in the priming-plus-reward condition [*t*(19) = −5.26, *p*<0.001]. Conversely, MVC in the “post” phase was decreased in the “baseline” phase in the control [*t*(19)  = 4.60, *p* = 0.002] and priming [*t*(19)  = 2.81, *p* = 0.011] conditions (Fig. 4). Although we do not know the exact reason for this, it is conceivable that three trials of a 5-s MVC with a 6-s inter-trial interval could induce cumulative intertrial fatigue [13], resulting in a decrease in MVC in the “post” phase. In fact, the 3^rd^ MVC was significantly decreased compared with the 1^st^ MVC in the “post” phase in the priming-plus-reward condition {one-way ANOVA [F_(1.458, 27.707)_  = 4.16; MSE = 338.7; *p* = 0.037; Effect size: η^2^ = 0.18] with Dunnett’s multiple comparison post hoc test (*p* = 0.015)}, and such a decrease was also observable in other cases in almost exactly the same manner.

Unconscious goal pursuit has been proposed to be produced by two processes, the preparation of action and the detection of a positive reward signal [12]. The former is associated with the perceptual, sensory, and motor level, which may be based on the ideomotor principle [12]. The latter is involved with subcortical regions such as the basal ganglia in the limbic structures, which play a role in reward processing and the recruitment of effort for action [5]. Moreover, the coactivaion of those processes may play a key role in the proposed mechanism of unconscious goal pursuit [2], which is supported by linear trends in motor performance (reaction time: correlation ratio = 0.90; rate of force: correlation ratio = 0.91; total effort: correlation ratio = 0.88) and in corticospinal excitability (MEP: correlation ratio = 0.87) among the three experimental conditions in our study.

Unconscious information processing in cognitive control and decision making are not only associated with subcortical regions such as the basal ganglia (more specifically in the ventral pallidum) and the thalamus [5], [14], and with motor-related brain areas (i.e., premotor and supplementary motor areas) [3], [4], but also with higher-level areas such as the anterior cingulate cortex [15], [16], [17]. Subliminal primes related to rewards processed in the ventral pallidum can motivate people to increase the effort they invest in behaviors, regardless of whether the stimulus is consciously visible [5]. Our MEP results indicate that barely visible priming with motivational reward actually alters the background state of the motor system, revealing that the positive reward signal attached to a goal can penetrate all the way up to the motor cortex, the final cortical stage of the motor execution program, with little consciousness. In other words, the affective-motivational effect on the motor system was enhanced by the positive stimulus-induced reward signal. This alteration helps to enhance not only the submaximal but also the maximal level of voluntary force exertion.

A pioneering study by Ikai and Steinhaus [7] proposed that MVC is limited by psychological inhibiting factors, based on experimental results showing that MVC was enhanced by manipulations such as the sound of a gunshot or a shout during maximal exertion efforts. We conclude that this psychological limiting factor can be removed or attenuated by manipulation of the barely conscious background state of the motor system by a positive reward signal attached to a primed goal. Such manipulation might also have practical value for those who are involved in various sporting activities or rehabilitation. The psychological limiting factors may be associated with the neural activity in one of the segmented basal ganglia-thalamocortical circuits, the limbic circuit that begins and ends in the anterior cingulate area and medial orbitofrontal cortex. However, we do not know to what exact extent that unconscious information processing involves in neural activity in the limbic circuit, a puzzle that remains to be solved.
